# Humour as emotion regulation and resource in autism: a narrative review

**DOI:** 10.3389/fpsyt.2026.1770298

**Published:** 2026-02-26

**Authors:** Mirella Manfredi, Pierrick Laulan, Andrea C. Samson

**Affiliations:** 1 Department of Child and Adolescent Psychiatry, University of Zurich, Zürich, Switzerland; 2 Faculty of Psychology, UniDistance Suisse, Brig, Switzerland; 3 Swiss Center for Affective Sciences, University of Geneva, Geneva, Switzerland; 4 Emotion and Memory Laboratory, Faculty of Psychology and Educational Sciences, University of Geneva, Geneva, Switzerland; 5 Center for Interdisciplinary Study of Gerontology and Vulnerability, University of Geneva, Geneva, Switzerland; 6 Department of Special Education, University of Fribourg, Fribourg, Switzerland

**Keywords:** autism, emotion regulation, humour, neurodiversity, well-being

## Abstract

Humour is closely linked to psychological well-being in neurotypical populations, supporting emotion regulation through mechanisms such as cognitive reappraisal, distraction, and positive affect. In autism, emotion regulation presents unique challenges, and humour use shows qualitative differences, including reduced reliance on adaptive strategies and a high prevalence of gelotophobia. This narrative review synthesises current evidence on humour as both a trait and a character strength. Findings suggest that although humour is less frequently employed in individuals with autism, it can act as a selective but valuable resource for resilience and well-being. Intervention studies, particularly those developed within neurodiversity-affirming frameworks, highlight the potential of humour-based approaches to strengthen emotional and social functioning in autism.

## 1 Introduction

Humour plays a fundamental role in both our psychological well-being and social functioning (1). Given its ubiquitous presence in human interaction, the capacity for humour can be regarded as a core component of the human experience itself (2). Despite the importance of humour in different areas of human experience and its relevance to multiple branches of psychology, humour has long remained relatively understudied. However, in the past several decades, a steady accumulation of research has focused on various aspects of humour (1, 3–5).

Such studies have revealed a positive association between a sense of humour and psychological well-being (6–9). In particular, it has been observed that high levels of a sense of humour are associated with low scores of depression, stress, anxiety, fear, and feelings of loneliness (10–13). In addition, other works have shown that a strong sense of humour is closely associated with a high positive effect on longevity and social functioning (14–16).

Moreover, several pieces of evidence suggest that humour may function as an effective form of emotion regulation, defined as “the attempts to influence what emotions we have, when we have them, and how we experience or express them” (17, p. 275). In a similar line, humour has been identified as an effective coping mechanism in situations of elevated stress (18). This particular function of humour has been extensively explored in studies involving individuals exposed to extreme adversity, such as survivors of concentration camps (e.g., 19). In these contexts, humour—often conveyed through jokes targeting oppressors or reflecting on shared hardships—plays a vital role in reinforcing group solidarity, enhancing self-esteem, promoting individual well-being, and preserving a sense of hope. Frequently characterised by incongruity, humour enables a shift in perspective that allows individuals to cognitively reframe stressful experiences, viewing them from a less threatening standpoint. Additionally, the positive emotions generated through humour may yield physiological advantages, contributing to psychophysiological health by facilitating recovery from the cardiovascular effects associated with stress-induced negative emotions (20).

Following this line, Carver et al. (21) examined a sample of 163 patients with early-stage breast cancer, whose psychosocial adjustment had initially been assessed during the year following surgery. The same patients completed the same assessments again, 5–13 years after the operation. Initial well-being outcomes were found to be relatively strong predictors of long-term well-being. Furthermore, the study showed that the use of humour was positively associated with optimism and that humour use predicted lower levels of distress in patients. These findings confirm that humour can help individuals regain a sense of control by redefining circumstances as less threatening.

The effect of humour on well-being has been documented primarily in neurotypical individuals; however, emotion regulation poses substantial challenges in other populations, particularly among individuals on the Autism Spectrum (AS) (22, 23). Autism is primarily presented as a neurodevelopmental condition characterised by difficulties in social communication and social interaction and by restricted, repetitive patterns of behaviours, interests, or activities (DSM-5; American Psychiatric Association [APA] 2013). Individuals within these populations frequently exhibit elevated rates of anxiety and depression, which can have profound effects on both the individuals themselves and their families (24, 25). Emotion regulation difficulties are common (26–29), potentially limiting the effective use of adaptive emotion regulation strategies, such as humour, to manage stress and reframe threatening situations (30). In light of these emotional vulnerabilities, identifying effective emotion regulation strategies is of critical importance.

This narrative review seeks to synthesise current knowledge on the role of humour in autism, highlighting existing research gaps, and ultimately informing the development of effective interventions aimed at enhancing emotional well-being and mitigating the risk of anxiety and depression in this population. This narrative review is structured in five main sections. The first section focuses on neurotypical individuals, outlining Gross’s emotion regulation process model and examining how humour may function within this framework. The second section continues to address neurotypical populations by presenting empirical evidence on the regulatory mechanisms of humour, particularly its role as a cognitive reappraisal strategy, strategy linked to attentional deployment (distraction), and source of positive affect, and by comparing the effects of positive versus negative humour. In the third section, again in the neurotypical context, humour is conceptualised as a psychological trait and character strength, with a particular emphasis on its relationship with personality traits and emotional well-being. The fourth section shifts the focus to individuals with autism spectrum disorders, applying the extended process model to highlight the unique challenges of emotion regulation and exploring how humour is used, in different and selective ways, as a regulatory strategy in individuals with autism. The fifth section continues to focus on autism by examining humour as a character strength in populations on the AS, discussing related constructs such as gelotophobia, and reviewing intervention studies aimed at improving emotional well-being through humour within both conventional and neurodiversity paradigms.

The final sections examine humour-based approaches in autism by reviewing intervention studies, integrating neurobiological and genetic perspectives on experience-dependent plasticity, and translating this evidence into practical, neurodiversity-affirming clinical guidance for the use of humour in emotion regulation.

## 2 Methods

Consistent with best-practice guidelines for narrative reviews (31), we combined targeted keyword searches with backward and forward citation tracking. Searches were conducted in PsycINFO, PubMed, Web of Science and Scopus between January 2025 and May 2025, using combinations of terms for *humour/humor*, *autism/ASD, AS*, *emotion regulation*, *intervention*, and *character strength*. Inclusion criteria were (a) empirical studies or systematic reviews published in peer-reviewed journals, (b) written in English or French, and (c) reporting quantitative or qualitative data on humour perception, production, or usage in individuals on the autism spectrum. Exclusion criteria were grey literature, single-case reports, and studies focusing exclusively on comic strips or irony comprehension without an emotion-regulation component.

## 3 Results

### 3.1 Emotion regulation strategy in neurotypical individuals

The process model of emotion regulation proposed by Gross (1998, 2015) conceptualises emotion regulation as a multi-phase sequence comprising four key stages: *identification*, *selection*, *implementation*, and *monitoring*. The process begins when an emotion captures attention, prompting the individual to engage in goal-setting and to make conscious or unconscious decisions about whether the current or anticipated emotional state should be modified—either amplified, diminished, or sustained (identification stage). The next step involves selecting an appropriate regulatory strategy (selection stage), followed by determining and enacting the specific behaviours or cognitive approaches required to apply that strategy (implementation stage). The final phase entails evaluating the effectiveness of the chosen strategy (monitoring stage), which informs whether to continue, adjust, or discontinue its use.

### 3.2 Humour as an emotion regulation strategy in neurotypical individuals

The process described in this model can be applied to humour (32), which, as previously mentioned, allows to regulate emotions to deal with stressful situations. Humour can be considered an active and effective strategy for regulating emotions and achieving emotional well-being. Following this, we consider both positive and negative forms of humour, exploring how different types of humour may produce distinct psychological and physiological effects.

The underlying cognitive mechanisms include cognitive change, particularly cognitive reappraisal and perspective shifting, which are closely associated with the detection and resolution of incongruity (33), as well as engagement with absurd or unexpected elements. Humour may also function through distraction and psychological distancing, allowing individuals to detach from distressing thoughts or emotions (32). Additionally, the experience of humour typically elicits positive emotional states such as mirth, exhilaration, and laughter, which may in turn be accompanied by physiological changes—for example, reductions in cortisol levels (34).

From this perspective, some researchers have focused on the positive effects of humour through cognitive change mechanism (35), others have explored its role as a cognitive distraction (36), and still others have examined it in the context of eliciting positive emotions (37). We will summarise the main results of these previous studies below.

Samson et al. (38) investigated humour as an emotion regulation strategy tapping into the cognitive change mechanisms associated with strategies such as cognitive reappraisal. In this study, the efficacy of serious (conventional) reappraisal was compared to humorous reappraisal. Participants were exposed to aversive images and instructed to respond either with humorous reappraisal, serious reappraisal, or simply by attending to the images without regulating their emotions. The results showed that although humorous reappraisal was rated as more cognitively demanding and less often successfully applied, it proved more effective than serious reappraisal in reducing negative emotions and increasing positive ones. Interestingly, one week later, the effect on negative emotions was still present–pictures that were reappraised humorously before were perceived as less negative–while the differential effects on positive emotions disappeared. The authors suggested that the exaggerated shift in perspective and the positive affect elicited by humour may contribute to its regulatory power on the short term, while the effects to down-regulate negative emotions persist even longer. Similarly, Kugler and Kuhbandner (39) explored humorous reappraisal, demonstrating that reinterpreting negative situations humorously not only significantly reduced subjective emotional intensity but also preserved memory recognition. This suggests humour’s unique regulatory benefit in balancing immediate emotional relief and cognitive processing of negative experiences.

Within this framework, humour has shown promise for individuals vulnerable to affective disorders. Braniecka et al. (40) observed that patients in remission from depression effectively used humour-based reappraisal to reduce negative affect and increase positive emotional states. Humour not only facilitated psychological distancing but also proved comparably effective to traditional positive cognitive reappraisal strategies, supporting its integration into therapeutic interventions aimed at enhancing emotional resilience. However, in a more recent study, the same authors (41) compared two distinct humour-based strategies: stress-related humour and non-stress-related humour. Outpatients with depression in remission participated in a randomised experiment that evoked personal stress and the subsequent application of either stress-related humour, non-stress-related humour, or a non-humorous regulation strategy. Unlike the non-humorous regulation, the humour-based strategies had adaptive consequences, both immediately and over time; however, the non-stress-related humour proved to be the most beneficial and the only effective strategy in the presence of attention deficits. Based on these findings, we could speculate that humour related to the stressor is similar to humorous reappraisal, while humour unrelated to the stressor acts as a cognitive distraction.

Strick et al. (36) investigated the effects of humour on mood improvement by conceptualising humour as a *cognitive distractor*. Based on Van Dillen and Koole (42) working memory distraction model, the authors hypothesised that the cognitive demands involved in humour processing, particularly incongruity resolution, may reduce negative emotions more effectively than equally positive but non-humorous stimuli. Participants in their study viewed neutral, slightly negative, and strongly negative images, followed by humorous or non-humorous positive stimuli. Humour led to significantly lower negative affect in both slightly negative and strongly negative conditions. Further analyses showed that the more cognitively demanding the humorous stimuli were, the more effective they were in attenuating negative emotions. Interestingly, Harm et al. (43) found humour particularly effective as an extrinsic emotion regulation strategy across age groups, reinforcing its broad applicability and suggesting that humour’s regulatory benefit extends beyond mere cognitive distraction, perhaps involving additional affective processes.

Crawford and Caltabiano (37) analysed whether humour can generate *positive emotions* and contribute to improving emotional well-being. The researchers recruited 55 adults, divided into three groups: one group participated in an eight-week humour skills training program; a second group met for weekly social gatherings without specifically humorous content; and a third group received no intervention at all. Participants were assessed at the beginning, at the end of the program, and three months later, using standardised psychometric tools measuring positive and negative affect, optimism, sense of control, self-efficacy, and levels of anxiety, depression, and stress. The results showed that only the group that followed the humour skills program reported significant improvements. Specifically, there was an increase in self-efficacy, optimism, perceived emotional control, and positive emotions. At the same time, levels of perceived stress, anxiety, depression, and negative affect decreased. These changes were sustained over time, with positive effects still evident three months after the program concluded. The other two groups, in contrast, did not show significant improvements. The study is based on Barbara Fredrickson’s “Broaden-and-Build” theory (44), which posits that positive emotions broaden individuals’ cognitive and behavioural resources, enhancing resilience and the ability to cope with adversity. According to the authors, humour fits perfectly within this framework.

#### 3.2.1 Positive and negative humour to regulate emotions

Other works investigated and compared the impact on positive versus negative humour on emotion regulation. In this regard, Samson and Gross (32) proposed three hypotheses regarding the psychological mechanisms underlying the effectiveness of different types of humour. *First*, they hypothesised that positive humour—characterised as benevolent, tolerant, and self-ironic—is more effective than negative humour, which is hostile, sarcastic, or disparaging. *Second*, they predict that positive humour compared to negative humour is more successful in both reducing negative emotions and enhancing positive ones. *Third*, they suggest that the greater effectiveness of positive humour cannot be explained by differences in task difficulty, participants’ expectations, or social desirability. To verify these hypotheses, the authors investigated the use of humour as a strategy for emotion regulation, with a specific focus on the distinction between positive (benevolent) and negative (disparaging) humour. Through two experimental studies, the authors demonstrated that positive humour is significantly more effective than negative humour in decreasing negative emotions and enhancing positive ones. These effects are not attributable to confounding variables such as task difficulty or social desirability. The practical implications suggest that fostering positive humour may promote emotional well-being and resilience, and could be effectively integrated into clinical, educational, and organisational settings. In contrast, negative humour may be detrimental or beneficial only under extreme circumstances.

#### 3.2.2 Humour and emotion regulation: insight from neuroimaging and physiological data

Recent neuroimaging studies offer insights into the neural substrates of humour reappraisal. Wu et al. (45) employed functional magnetic resonance imaging (fMRI) to identify distinct neural networks activated during the humorous reappraisal of negative stimuli. Their findings revealed enhanced activation in a hippocampus-thalamus-prefrontal network linked to cognitive reappraisal (“aha” moment), alongside amygdala-cerebellum circuits associated with emotional release and laughter (“haha” moment). Stronger hippocampo-prefrontal connectivity was positively correlated with improved emotional outcomes, indicating humour’s capacity to simultaneously modulate cognitive and affective components of emotion regulation.

Complementing neural evidence, physiological studies reinforce humour’s stress-buffering effects. Froehlich et al. (46) demonstrated that brief humorous interventions prior to acute stress exposure significantly reduced subjective stress ratings and cortisol levels without impairing cognitive performance. These results provide biological validation of humour as an effective, quick-acting protective mechanism against stress-induced physiological arousal.

Taken together, empirical evidence discussed so far, from behavioural experiments, neuroimaging, and physiological studies underscores humour’s robust effectiveness as a multidimensional emotion regulation strategy. Positive humour, particularly through cognitive reappraisal, emerges as uniquely potent in mitigating negative emotional states, promoting emotional resilience, and fostering psychological well-being across diverse populations. In the following two sections, we will describe research that has conceptualised humour as a trait and humour as a character strength.

### 3.3 Humour as a psychological trait in neurotypical individuals

As Martin and Ford (1) noted, a sense of humour can be conceptualised as a personality trait or set of traits encompassing the perception, production, and appreciation of humour, as well as its use in everyday life. Humour expression varies across individuals and is systematically related to broader personality traits. For example, Di Fabio et al. (47), using network analysis in a large sample of Italian workers, showed that self-enhancing humour was the most central humour style and was positively associated with emotion control, whereas self-defeating humour was negatively associated with this facet. Aggressive humour emerged as a key bridge node linking humour styles and personality, highlighting the relevance of humour for psychological well-being.

Research on humour has led to the development of several instruments assessing individual differences in humour perception and production. Widely used measures include the Sense of Humor Questionnaire (SHQ; 48), the Multidimensional Sense of Humor Scale (MSHS; 49), the Situational Humor Response Questionnaire (SHRQ; 50), and the Sense of Humour Scale (SHS; 51). The 3WD questionnaire further distinguishes preferences for incongruity-resolution, nonsense, and sexual humour and relates them to personality traits such as extraversion and neuroticism.

Although humour has often been conceptualised as an inherently adaptive trait, individuals do not always use humour in psychologically or socially beneficial ways (1). This observation led to the distinction of four humour styles: affiliative humour, which promotes social bonding; self-enhancing humour, which supports coping and emotional regulation; aggressive humour, characterised by sarcasm and ridicule; and self-defeating humour, involving self-directed disparagement (52). These styles differ markedly in their associations with well-being, with affiliative and self-enhancing humour generally linked to positive outcomes, and aggressive and self-defeating humour associated with poorer psychological adjustment.

The Humour Styles Questionnaire (HSQ; 52) was developed to assess these four styles and remains the most widely used instrument in this area. Related tools include the Coping Humour Scale (CHS; 50), which captures humour use in response to stress but also overlaps with maladaptive styles, and the State-Trait-Cheerfulness-Inventory (STCI; 53), which distinguishes between momentary and dispositional cheerfulness. Together, these measures provide a robust framework for investigating the role of humour in well-being, resilience, and interpersonal functioning, as further supported by work using the Comic Style Markers (54).

### 3.4 Humour as a character strength

Within the framework of Positive Psychology, the Values in Action (VIA) Classification of Strengths was developed by Peterson and Seligman (55) as a taxonomy of positive traits contributing to a meaningful life. The classification includes 24 character strengths grouped into six core virtues, with humour conceptualised as a morally valued strength that can foster virtue.

Empirical evidence consistently links humour as a character strength to life satisfaction and psychological well-being. In a large two-country study of U.S. and Swiss adults, humour showed a moderate positive association with life satisfaction (r = .28–.38) and emerged as a strong predictor of the pleasure-oriented pathway to happiness (56). These findings were replicated in a German validation of the VIA-IS, which demonstrated high internal consistency and good test–retest reliability for humour (57). Humour has also been associated with psychological resilience and effective stress management (1).

More fine-grained analyses within the VIA framework indicate that humour as a character strength is primarily linked to prosocial and affiliative humour styles (58). However, its effects on well-being vary depending on humour style, as shown by comparisons between the VIA-IS humour scale and the Humour Styles Questionnaire (HSQ; 52), which distinguishes adaptive and maladaptive humour styles (59).

Research in older adults further supports the role of humour in well-being, while highlighting age-related changes. Large-scale studies show that humour is positively associated with life satisfaction, particularly with pleasure and engagement, although its association with meaning weakens in later life (60). Humour may also function as an adaptive coping strategy in aging, helping individuals manage stress and maintain quality of life, underscoring its relevance for positive psychology and intervention development in older populations (61, 62).

### 3.5 Emotion regulation strategy in autism

Emotion regulation represents a critical and often impaired area in individuals with autism, negatively affecting their quality of life and social integration. According to Mazefsky et al. (23), emotion dysregulation in autism stems from a complex interaction between neurobiological differences in emotional circuitry and core autism characteristics, creating unique regulatory challenges that require specialised intervention approaches. The non-systematic review by (63) provides an overview of the main challenges in emotion regulation among individuals with autism, analysed through the lens of Gross’s extended process model. In the identification phase, many individuals with autism experience difficulties recognising and understanding their own emotions, often linked to the presence of alexithymia, which is significantly more prevalent in populations on the AS compared to neurotypical groups (64). However, even in the absence of alexithymia, challenges persist due to maladaptive beliefs about emotions and a tendency toward emotional inertia (65, 66). During the selection phase, evidence suggests that individuals with autism tend to employ less sophisticated and more behaviourally-oriented strategies (e.g., avoidance) rather than cognitively demanding approaches like reappraisal. This pattern may be attributable to heightened emotional arousal and executive function impairments (67, 68). In the implementation phase, although individuals with autism are less likely to spontaneously use cognitive reappraisal, they have demonstrated the capacity to adopt such strategies when provided with guidance and practice. Notably, they may rely on alternative neural mechanisms during implementation (69, 70). Finally, in the monitoring phase, difficulties appear to be associated with reduced interoceptive awareness and psychological rigidity, which can hinder the flexible adjustment of strategies over time (71, 72). Overall, the authors emphasize the need for individualised, temporally sensitive approaches to better understand and support the emotion regulation processes in individuals with autism.

#### 3.5.1 Developmental trajectories of emotion regulation in autism

Research on emotion regulation in autism spectrum populations reveals distinctive and stable patterns of emotional processing that diverge from neurotypical development. Children on the autism spectrum tend to exhibit fragmented emotion regulation profiles, with significant difficulties in emotional awareness and processing (73), which are closely linked to higher rates of internalising symptoms such as depression and rumination (22, 63, 74).

Research consistently indicates greater dysregulation and less effective regulation overall. Compared to typically developing peers, children with autism show a different ER profile, characterised by less use of adaptive strategies (e.g., cognitive reappraisal, acceptance) and, at times, greater reliance on maladaptive strategies (e.g., expressive suppression, avoidance, venting). They also depend more heavily on caregivers, reflecting delays in the transition from co-regulation to self-regulation. These difficulties in ER are not only related to the severity of autism symptoms, executive functions, and language abilities, but also to social functioning (22).

Notably, while positive emotion regulation strategies generally reduce depressive symptoms in neurotypical children, they appear less effective in children with autism, suggesting qualitative rather than quantitative differences in emotional mechanisms. These regulatory differences are also critical to social development. Evidence indicates that effective use of cognitive reappraisal is associated with fewer autism-related symptoms and improved social functioning in children on the spectrum, whereas emotional suppression shows no such link (75). Emotion regulation thus plays a mediating role between internal emotional dynamics and external social behaviours. Longitudinal findings further show that emotion regulation difficulties emerge early and remain consistent over time, predicting a range of behavioural and social difficulties independent of cognitive ability (30, 76).

### 3.6 Humour as an emotion regulation strategy in autism

While much research has focused primarily on regulating negative emotions, subsequent studies have also examined how individuals with AS regulate positive emotions. A comprehensive systematic review documented this significant research gap in emotion regulation within autism (26). The ability to maintain and enhance positive emotional states contributes substantially to psychological well-being, yet remains peripherally addressed in the literature despite its clinical importance for populations on the AS. In 2015 (77), Samson and colleagues investigated how individuals on the AS regulate their emotions in comparison to neurotypical individuals. The study focused on three emotional domains—anger, anxiety, and amusement—and analysed data gathered from parental interviews and self-reported daily diaries completed by children. Findings revealed that individuals with autism tend to experience higher levels of anger and anxiety, and report lower levels of amusement compared to neurotypical individuals. However, while parents noted clear differences in the emotional experiences of their children on the AS, the self-perceptions of the participants with autism showed fewer discrepancies from neurotypical individuals regarding anxiety and anger. This divergence may stem from limited emotional awareness, a characteristic frequently observed in AS, or from challenges in accurately self-assessing one’s emotional states. Regarding emotion regulation, the study found that AS individuals use adaptive strategies—such as problem-solving, cognitive reappraisal, and social support—less frequently than neurotypical individuals. Instead, they rely more heavily on strategies traditionally classified as maladaptive from a neurotypical perspective, including repetitive behaviours and avoidance.

Interestingly, the frequent use of repetitive behaviours suggests that these may serve a regulatory function, helping to manage both negative and positive emotions (29). While conventional clinical paradigms have historically classified these behaviours as maladaptive and limitations that constrain emotional and social development, emerging research frameworks fundamentally reorient this understanding. The recently proposed neural-preferencing locus of control model (78) offers an important reconceptualisation of these regulatory patterns. This framework suggests that what has been labelled as maladaptive regulation from a neurotypical perspective may actually represent functional internal control mechanisms within autistic neurocognitive organisation. According to this model, repetitive behaviours and avoidance strategies may serve as effective ways for individuals with AS to maintain neurobiological homeostasis when faced with overwhelming environmental demands. This perspective aligns with emerging neurodiversity-affirming approaches that recognise different, rather than deficient, patterns of emotional processing in autism.

In this context, amusement is a positive emotion that plays a crucial role in social bonding. A particularly noteworthy aspect of Samson et al. (68) study concerns the regulation of this emotion. Individuals on the AS not only experienced less amusement overall but also employed different strategies when attempting to regulate this emotion. These differences in processing humour and modulating positive emotional experiences have significant implications for social interaction and quality of life. This understanding is further enriched by a recent systematic review that examined humor in AS by analysing 43 articles on the topic (79). After a rigorous selection process, the researchers identified specific patterns in humor processing among individuals with AS. Their findings indicate that while individuals with AS can generally detect humor in audiovisual and written formats, they exhibit specific comprehension difficulties that vary according to presentation modality (79). In particular, individuals with AS (ranging from children to adults in the studies analysed) demonstrate more pronounced difficulties with written humor, pure auditory humor stimuli, and non-verbal humorous drawings, while showing relative strengths in multimodal humor processing. These variations in humor processing across different sensory channels may contribute to the observed challenges in regulating positive emotional experiences such as amusement.

In a subsequent study published in 2022, Samson and colleagues explored the use of emotion regulation strategies among individuals on the AS, Williams syndrome (WS), and intellectual disability (ID), aiming to examine group differences and their associations with anxiety. Using data from a parent-reported survey conducted during the early months of the COVID-19 pandemic, the researchers analysed responses from 785 individuals on the AS and intellectual disability (ID), 596 on the AS without ID, 261 with Williams syndrome (WS), and 649 with unspecified ID. Multilevel analyses revealed that strategies such as rumination, information avoidance, and repetitive behaviours were associated with higher levels of anxiety, while focusing on positive aspects was linked to lower anxiety levels. Interestingly, only individuals with autism without ID were reported using humor more frequently, and they also experienced lower levels of anxiety compared to other groups. This study underscores the central role of emotion regulation in neurodevelopmental conditions and suggests that adaptive strategies, such as focusing on the positive, may contribute to reduced anxiety. However, further research is needed to deepen our understanding of these dynamics and to develop tailored interventions that promote emotional well-being across AS, WS, and ID populations.

In the same year, Nagase examined the relationship between autistic traits and the use of humor as an emotion regulation strategy in the general population. Participants completed questionnaires assessing social responsiveness and the use of humour in coping with interpersonal stress. The results showed that while the overall level of autistic traits was not significantly related to humour use for emotion regulation, two specific traits were. Reciprocal social communication abilities were negatively associated with humour use, whereas restricted interests and repetitive behaviours showed a positive association. These findings suggest that different aspects of autistic traits may influence the use of humour in distinct ways when it comes to emotion regulation. The study is particularly valuable in offering new perspectives on how individuals with autistic traits may benefit from adaptive emotion regulation strategies. The results may inform intervention efforts aimed at enhancing emotional coping through humour, especially for individuals who struggle with social communication but exhibit a propensity for repetitive and focused behaviours.

Research by Samson and others (80) highlights that individuals on the AS, particularly those without ID, tend to experience less amusement than neurotypical peers and have difficulty regulating positive emotions through humour. These challenges are linked to difficulties in processing humour and sharing playful moments, which may contribute to social isolation. However, some individuals with autism use humour as an adaptive emotion regulation strategy, and those who do report lower levels of anxiety, suggesting a potentially protective role. Nagase’s study further showed that in the general population, specific autistic traits influence humour use differentially: difficulties in social communication reduce its use, while restricted interests and repetitive behaviours are positively associated with it. Together, these findings suggest that humour can be a selective but valuable resource for emotion regulation and may be beneficial to emphasise in targeted interventions aimed at improving emotion regulation and social well-being in individuals with autism.

### 3.7 Humour as a psychological trait and character strength in autism

As mentioned above, humour is recognised as one of the 24 character strengths in the VIA classification, and it is associated with psychological well-being, positive social relationships, and resilience (81). However, among individuals with autism, this strength appears to be less frequently utilised compared to the neurotypical population. Studies conducted on adults with autism have found that humour ranks among the least endorsed strengths, for instance occupying the 15th position out of 24, whereas in neurotypical controls it ranks significantly higher, in second place (82). While humour is generally perceived as a source of enjoyment (life of enjoyment), in individuals with autism, it does not seem to contribute meaningfully to a life of purpose and social engagement (life of meaning and commitment). Moreover, humour tends to be an underutilised interpersonal and emotional strength in the population on the AS, in contrast to more commonly endorsed intellectual strengths such as love of learning or honesty (83, 84). Nevertheless, caregivers, family members, and individuals with autism themselves often identify humour as a positive strength, even though it is rarely emphasised in intervention programs (85).

Some questionnaires related to humour were also administered to individuals on the AS or to their family members. In particular, some questionnaires tested a related condition concerning humour that is often observed in individuals on the autism spectrum: *gelotophobia*. It is the fear of being laughed at (from the ancient Greek gelos, which means “laughter” and phobos, which means “fear”) and is associated with the tendency to interpret others’ laughter as if it were aimed towards oneself, feeling ashamed and ridiculed as a consequence (60). In adults, gelotophobia is typically assessed using the PhoPhiKat-45 (short version: PhoPhiKat-30), a self-report questionnaire that measures three humor-related dispositions: fear of being laughed at (gelotophobia), enjoyment of being laughed at (gelotophilia), and enjoyment of laughing at others (katagelasticism) (60). These instruments have been used in several studies investigating gelotophobia in adults with autism, revealing markedly higher prevalence rates compared to neurotypical peers. For example, Samson et al. (86) examined the levels of gelotophobia in relation to remembered experiences of being laughed at in the past in individuals on the AS. Approximately 45% of individuals on the AS, but only 6% of controls, had at least a mild form of gelotophobia. Gelotophobia was correlated with the frequency and severity of remembered teasing and mockery in the past. Furthermore, individuals on the AS are less able to laugh at themselves (gelotophilia) but enjoy laughing at others (katagelasticism) to the same extent as controls. Similarly, (Leader et al. (87) found that 87.4% of high-functioning adults on the AS scored above the cut-off for gelotophobia, compared to 22.6% in a neurotypical comparison group. Weiss et al. (88) reported that 72.2% of adults with autism met the threshold for gelotophobia, in contrast to only 25% of neurotypical participants. These findings suggest that gelotophobia in adulthood is strongly associated with emotional vulnerability factors such as social anxiety, low self-esteem, and difficulties in emotion regulation. For children and adolescents, a parent-report version, PhoPhiKat-30c, has been developed (89). This version adapts the 10 gelotophobia items into third-person statements that describe observable behaviours. Treichel et al. (90) applied this version in a study involving children, adolescents, and young adults (ages 5–25) diagnosed with autism, WS, or Down syndrome. The results revealed that approximately 60% of participants with autism showed at least mild levels of gelotophobia, whereas only around 6–7% of individuals with WS or Down syndrome met the same threshold. Importantly, the study found that gelotophobia levels were not solely predicted by diagnostic category. Instead, individual temperamental traits, particularly high seriousness and bad mood, emerged as the strongest predictors of gelotophobic tendencies.

In 2023, Treichel et al. employed the State-Trait Cheerfulness Inventory (STCI) to examine the relationship between humor-related temperament and gelotophobia, finding that children with autism and adolescents, compared to peers with Down syndrome and WS, showed significantly higher levels of seriousness and bad mood, as well as lower levels of cheerfulness. Regression analysis revealed that this temperamental profile was the strongest predictor of the fear of being laughed at, surpassing diagnostic group membership, suggesting that dispositional traits may play a critical role in the development of gelotophobia.

A further perspective is provided by the study of Samson and Antonelli (82), which utilised the Satisfaction With Life Scale (SWLS) and the Orientation to Happiness Scale (OHS) to assess the contribution of humour to life satisfaction in adults. Although no significant differences emerged between the AS group and neurotypical controls in overall life satisfaction scores, humour was positively correlated with well-being only in the neurotypical comparison group. This suggests that, for individuals with autism, humour does not function as an equivalent psychological resource. Finally, Wu et al. (91) administered the HSQ to a large school-based sample, finding that participants with autism displayed a lower inclination toward affiliative and self-enhancing humour styles, as well as significantly reduced use of self-defeating humour. In contrast, their use of aggressive humour was comparable to that of their neurotypical peers.

### 3.8 Humour-targeting interventions

Humour has been successfully used among typically developing adolescents and adults (92, 93) and in clinical populations (94–97) as an intervention to reduce anxiety and stress and increase well-being. Recent advances in cognitive neuroscience have contributed to a more nuanced understanding of the neural architecture associated with autism. Neuroimaging and electrophysiological studies have documented atypical patterns of functional connectivity and processing style, which are increasingly interpreted not as indicators of deficit, but as reflecting alternative neurodevelopmental trajectories (98). These findings have coincided with broader epistemological shifts within autism research, prompted in part by sustained advocacy from the autistic community and critical reassessments within the academic field. One of the most significant of these shifts is the emergence of the neurodiversity paradigm, which conceptualises autism as a natural variation within the spectrum of human cognitive functioning (e.g., 99, 100). In contrast to traditional medicalised models, which aim to remediate core features of autism, this framework emphasises heterogeneity, self-determination, and the identification of cognitive and affective strengths. It posits that the lives of individuals on the autism spectrum can be rich and fulfilling on their own terms, rather than defined by deviation from normative developmental paths. As a consequence, research framed within this paradigm prioritises subjective well-being, contextual adaptability, and ecological validity over symptom reduction alone (101).

The study by Nocon et al. (84) is grounded in this perspective. It evaluates a positive psychology intervention targeting humour, conducted with a sample of adults with autism. Participants first completed a self-assessment of character strengths, identifying traits such as honesty, fairness, kindness, appreciation of beauty, and love of learning, i.e. attributes frequently endorsed in autistic self-narratives (102). A subset of participants then engaged in the *Three Funny Things* exercise, which involves documenting three humorous experiences each day over a one-week period (103). Post-intervention outcomes indicated a modest reduction in depressive symptoms, with no significant change in self-reported happiness. These results, while preliminary, suggest that humour, often underutilised as an emotional or interpersonal resource in populations on the AS, can nonetheless be cultivated through structured, low-intensity interventions. The study also illustrates the feasibility of designing wellbeing-focused interventions that centre individual agency and self-identified strengths, rather than normative social conformity. These findings align with previous research in younger populations.

Wu et al. (104), for example, developed a humour-based intervention in which adolescents with autism were exposed to complex humorous material drawn from articles and images. Following the intervention, a significant improvement was observed exclusively in the comprehension and appreciation of one specific type of humour—nonsense humour—among adolescents on the autism spectrum. Additionally, an increased use of affiliative humour, a style associated with prosocial interaction, was reported. While these findings are both promising and distinctive, no comparable improvements were noted in the understanding of incongruity-resolution humour, and the effects of the intervention were not evaluated beyond the immediate post-training phase. The authors of the study adopted a conventional framework, grounded in the assumption that interventions for individuals on the autism spectrum should aim to realign behaviour and underlying neurocognitive processes with normative developmental trajectories. It would be desirable for emerging therapeutic approaches employing humour in autism interventions to instead focus on strengthening and valuing the unique sense of humour intrinsic to individuals on the spectrum (99).

Together, empirical research on emotion regulation in individuals on the autism spectrum consistently reveals distinctive developmental trajectories, characterised by a reduced reliance on adaptive strategies and a greater dependence on avoidance or repetitive behaviours. Experimental studies on humour in autism confirm that humour processing and the regulation of amusement follow qualitatively different patterns compared to neurotypical profiles, with relative strengths in certain modalities but persistent comprehension difficulties in others. Dispositional studies and those examining humour as a character strength further indicate that humour is less frequently valued or spontaneously employed by individuals with autism, and that related constructs such as gelotophobia show a high prevalence, potentially constraining the social and regulatory benefits of humour. At the same time, intervention research, although still limited, demonstrates that humour-related competences can be enhanced, both within traditional developmental frameworks and through approaches that embrace the neurodiversity paradigm. Taken together, this body of evidence suggests that humour in autism may be best understood not as a deficit, but as a selective and context-dependent resource that, when adequately supported, can fulfil important regulatory, interpersonal, and resilience-building functions.

### 3.9 Circuits and genes in humour-based regulation

At the genetic and neurodevelopmental levels, the goal is not to imply that psychosocial interventions alter genetic risk, but to situate intervention effects within experience-dependent plasticity. Integrative work in autism genetics emphasises substantial heterogeneity alongside convergence on pathways relevant to synaptic development and plasticity (105–107). Activity-dependent neuronal signalling provides a particularly useful bridge: neuronal activity regulates synapse development and plasticity via molecular cascades that influence transcription and local protein synthesis, and many autism-relevant genes participate in these activity-dependent networks (108). From this perspective, humour-based reappraisal can be conceptualised as a structured form of environmental input that repeatedly co-activates control and affective systems (and, when social humour is involved, socio-cognitive systems). A biologically grounded, testable hypothesis is therefore that effective humour-based regulation training may be associated with measurable changes in regulation-relevant outcomes (e.g., improved monitoring/adjustment, reduced stress reactivity) and, where feasible, task-related changes in connectivity within fronto-limbic and fronto-temporal systems.

## 4 Clinical translation: practical guidance for humour-based emotion regulation in autism

The clinical aim of humour-based emotion regulation is not to teach individuals to “be funny,” but to broaden their regulatory repertoire by using humour to support cognitive flexibility, psychological distance, and positive affect (32). First, clinicians should clarify regulatory goals (e.g., reducing emotional escalation, supporting recovery or social engagement) and screen for risk factors that may render humour aversive or unsafe, including gelotophobia, social anxiety, and a history of bullying or teasing (86, 109).

Second, it is important to identify the individual’s humour profile, including preferred modalities (visual/situational vs. verbal), social comfort, and humour styles (e.g., affiliative or self-enhancing vs. aggressive or self-defeating), using clinical interviews and, when appropriate, standardised measures (52). When alexithymia is prominent, brief emotion-awareness support may be required, as effective monitoring depends on recognising internal cues (64).

Third, intervention should begin with low-demand, positive-affect exercises that allow access to amusement without social performance pressure, such as developmentally adapted versions of brief positive psychology tasks (e.g., “Three Funny Things”; 103) and, more broadly, strengths-based approaches that can be tailored to autistic adults (84).

Fourth, humorous reappraisal could be taught in a structured manner as a variant of cognitive reappraisal, focusing on generating benign, non-hostile reinterpretations of mildly negative situations before generalising to more salient stressors. Evidence indicates that autistic adults can successfully implement instructed reappraisal, particularly when supported by explicit instruction and guided practice examples that reduce cognitive demands (69).

Finally, explicit safety rules are essential in social contexts, prioritising affiliative and self-enhancing humour, prohibiting ridicule or sarcasm directed at others, and requiring explicit consent. Feasibility and outcomes should be monitored continuously, assessing not only symptom change but also whether humour reduces arousal or, conversely, increases confusion or social threat, with the protocol adapted accordingly (110).

## 5 Limitations

Despite the aim of providing a broad and integrated overview of the use of humour as an emotion regulation strategy and its potential as a resource for clinical interventions in the context of autism, this work presents several limitations that should be acknowledged. First, as a narrative rather than a systematic review, the selection of studies, although guided by explicit criteria, does not ensure comprehensive coverage of the existing literature. Moreover, the absence of a structured evaluation of the methodological quality of the included studies exposes the review to potential selection and interpretation biases.

A second limitation concerns the marked heterogeneity of the included studies, which differ widely in terms of research design, participant characteristics (e.g., age, level of functioning, presence or absence of intellectual disability), the tools used to assess humor and emotion regulation, and the cultural contexts in which the studies were conducted. This variability limits the comparability of findings and complicates the formulation of generalizable conclusions.

Another limitation lies in the scarcity of longitudinal and experimental studies focusing on humor and autism. Much of the existing research is descriptive or cross-sectional, which restricts the ability to establish causal relationships between humour use and emotional or social outcomes. In addition, the long-term effectiveness of humour-based interventions remains largely unexplored.

It is also worth noting that most studies have primarily investigated verbal humour, often overlooking nonverbal, visual, or situational forms of humour that may be more accessible or preferred by some individuals with autism. Similarly, the literature is predominantly based on Western samples, with limited attention to cultural variations in humour perception and use.

Finally, although this review adopts a neurodiversity paradigm perspective, parts of the existing literature continue to frame differences in humour processing as deficits rather than as legitimate variations within the spectrum of human neurodiversity. In this regard, future studies should pay particular attention to the development and validation of humour-centred interventions that are culturally sensitive, accessible, and grounded in neurodiversity paradigm approaches. Such interventions should be co-designed with individuals with autism in order to address their actual needs, recognise and build upon their strengths, and promote emotional well-being and social participation. Integrating humour-based practices within educational, therapeutic, and community settings may help to strengthen emotional and interpersonal competencies without relying on normative models of behavioural conformity.

## 6 Conclusion

In conclusion, this review suggests that humour, albeit sometimes experienced and expressed differently than in neurotypical individuals, represents a potential strategy for emotion regulation in autism. Although differences in humour perception and use in autism present notable challenges, evidence suggests that positive forms of humour may also serve as adaptive resources for this population. Targeted interventions, developed within a neurodiversity-paradigm framework and co-designed with individuals with autism, may help strengthen emotional and social competencies, thereby enhancing quality of life. Further rigorous and interdisciplinary research will be essential to consolidate these findings and promote more inclusive approaches to mental health in both clinical and educational contexts.
